# *Erythrina mulungu* Alkaloids Are Potent Inhibitors of Neuronal Nicotinic Receptor Currents in Mammalian Cells

**DOI:** 10.1371/journal.pone.0082726

**Published:** 2013-12-13

**Authors:** Pedro Setti-Perdigão, Maria A. R. Serrano, Otávio A. Flausino, Vanderlan S. Bolzani, Marília Z. P. Guimarães, Newton G. Castro

**Affiliations:** 1 Laboratório de Farmacologia Molecular, Instituto de Ciências Biomédicas, Universidade Federal do Rio de Janeiro, Rio de Janeiro, Brazil; 2 Núcleo de Bioensaios, Biossíntese e Ecofisiologia de Produtos Naturais (NuBBE), Instituto de Química, Universidade Estadual Paulista Júlio de Mesquita Filho, Araraquara, Brazil; Dalhousie University, Canada

## Abstract

Crude extracts and three isolated alkaloids from *Erythrina mulungu* plants have shown anxiolytic effects in different animal models. We investigated whether these alkaloids could affect nicotinic acetylcholine receptors and if they are selective for different central nervous system (CNS) subtypes. Screening experiments were performed using a single concentration of the alkaloid co-applied with acetylcholine in whole cell patch-clamp recordings in three different cell models: (i) PC12 cells natively expressing α3* nicotinic acetylcholine receptors; (ii) cultured hippocampal neurons natively expressing α7* nicotinic acetylcholine receptors; and (iii) HEK 293 cells heterologoulsy expressing α4β2 nicotinic acetylcholine receptors. For all three receptors, the percent inhibition of acetylcholine-activated currents by (+)-11á-hydroxyerysotrine was the lowest, whereas (+)-erythravine and (+)-11á-hydroxyerythravine inhibited the currents to a greater extent. For the latter two substances, we obtained concentration-response curves with a pre-application protocol for the α7* and α4β2 nicotinic acetylcholine receptors. The IC_50_ obtained with (+)-erythravine and (+)-11á-hydroxyerythravine were 6 µM and 5 µM for the α7* receptors, and 13 nM and 4 nM for the α4β2 receptors, respectively. Our data suggest that these *Erythrina* alkaloids may exert their behavioral effects through inhibition of CNS nicotinic acetylcholine receptors, particularly the α4β2 subtype.

## Introduction

*Erythrina mulungu* (Papilionaceae) is a native tree from Southern Brazil, known as mulungu or coral tree due to its reddish flowers [1]. Tinctures and decoctions made from the leaves or barks of *E. mulungu* are often used in Brazilian traditional medicine as mild sedatives and to treat insomnia and depression [2]. Aqueous alcoholic extracts of *E. mulungu* produce anxiolytic-like effects in rats submitted to elevated T-maze and light/dark transition tests [3], [4], as well as antinociceptive and anticonvulsant effects in mice [5], [6], raising interest in the discovery of neuroactive compounds in the plant. Three previously known and one novel erythrinian alkaloid recently isolated from the flowers of *E. mulungu* reproduced some of the CNS effects of the polar extracts [7]–[10]. In particular, oral administration of (+)-erythravine and (+)-11α-hydroxyerythravine induced anxiolytic-like effects comparable to that of diazepam in mice, while (+)-11α-hydroxyerysotrine was only effective in some tests [8], [9]. Because these alkaloids were effective at low oral doses (3–10 mg/kg p.o.) that did not disrupt locomotion or exploratory activity, the authors suggested that the mechanism was non-GABAergic [8], but the molecular targets remained to be investigated.

**Figure 1:**
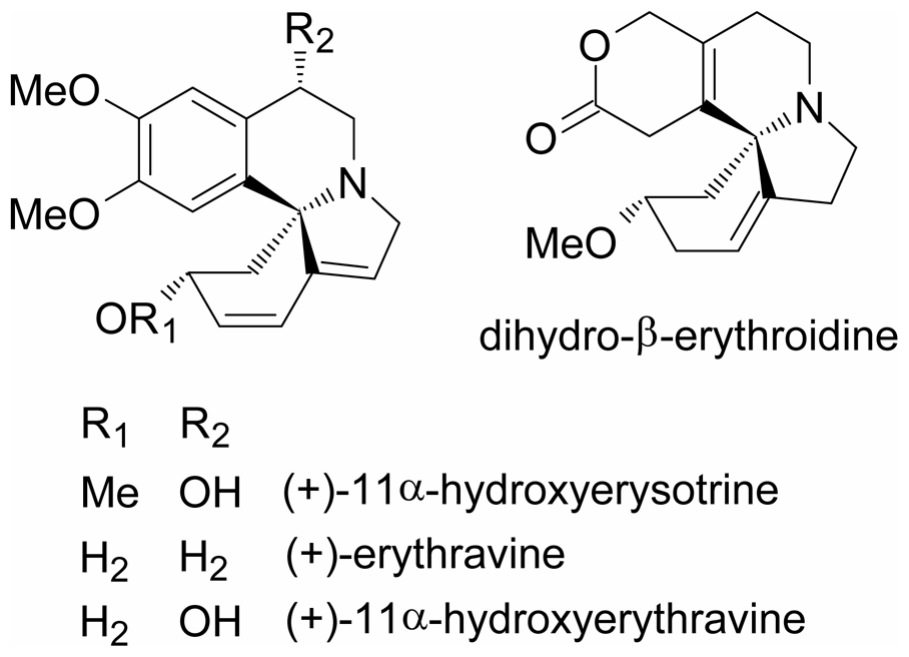
Structures of the alkaloids from *E. mulungu*.

There is accumulating evidence that changes in several neurotransmitter systems underlie anxiety disorders. The cholinergic system and particularly the nicotinic acetylcholine receptors modulate behavioral correlates of anxiety in the rat [11]–[14]. Nicotine and other nicotinic acetylcholine receptor agonists have complex anxiogenic effects that can be antagonized by dihydro-β-erythroidine (DHβE, Fig. 1) [11], [15]. This erythrinian alkaloid from *E. americana* is widely used as selective antagonist of neuronal α4β2 nicotinic receptors at nanomolar concentrations [16], [17]. Because the *E. mulungu* alkaloids are structurally related to DHβE and other erythrinian alkaloids known to act on nicotinic receptors [18], we investigated three orally active *E. mulungu* alkaloids (Fig. 1) as possible modulators of CNS nicotinic receptor channels using patch-clamp electrophysiology.

## Methods

### Ethics Statement

Animals were bred and treated in accordance with the NIH Guide for the Care and Use of Laboratory Animals. All experimental procedures were carried out in order to minimize animal suffering and were approved by the Ethics Committee on the Use of Animals of Universidade Federal do Rio de Janeiro, protocol n^o^ DFBICB029.

### Hippocampal neuron culture

Pregnant Wistar rats at 18 to 20 days of gestation were sacrificed under CO_2_ anesthesia, and hippocampal cells from the fetuses were isolated and cultured as described previously [19]. Usually 14 to 20 hippocampi were pooled, trypsinized, and mechanically dissociated, and then approximately 10^6^ cells were plated per 35-mm poly-l-lysine-coated dish. Cultures were maintained in a humidified atmosphere with 10% CO_2_ at 35°C. Maintenance medium was minimal essential medium (MEM) with Earle's salts (Invitrogen, Carlsbad, CA) supplemented with 5 g/l of d-glucose, 2 mM glutamine, 3.7 g/l NaHCO_3_, and 10% horse serum (HS, Gemini Bioproducts, Woodland, CA, USA or Laborclin, Pinhais, Brazil). Plating medium further contained 10% fetal bovine serum (FBS, Cultilab, Campinas, Brazil) and 20 µg/ml deoxyribonucleotidase type II and was progressively replaced by maintenance medium starting 1 or 2 days after plating. Cell proliferation was inhibited 6 to 7 days after plating, when the glial monolayer was confluent, by the addition of 14 µg/ml 5-fluoro-2'-deoxyuridine and 7 µg/ml uridine. Half of the medium was changed twice a week, and cultures were used 14 to 40 days after plating. Culture medium components were purchased from Invitrogen or Sigma (St. Louis, MO).

### PC12 cell culture

Wild-type rat pheochromocytoma PC12 cells (American Type Culture Collection ref. CRL-1721, USA) provided by Dr. S. S. Smaili (UNIFESP, Brazil) were grown at 37°C in a humidified incubator under 10% CO_2_ in RPMI 1640 medium with 5% FBS, 10% HS, 2 mM glutamine, 100 U/ml penicillin and 0.1 mg/ml streptomycin. Cells were plated in collagen-coated Petri dishes and maintained for at least three days before experiments.

### HEK 293 cell culture and transfection

HEK 293 cells (Banco de Células do Rio de Janeiro, Brazil) provided by Dr. J. P. B. Viola (INCA, Brazil) were grown at 37°C in a humidified incubator under 5% CO_2_ in minimal essential medium with 10% FBS, 2 mM glutamine, 100 U/ml penicillin and 0.1 mg/ml streptomycin. Cells were plated in Petri dishes and maintained for at least three days before experiments. Genes for the rat α4 and β2 (GeneBank L31620 and L31622, respectively) nicotinic acetylcholine receptor subunits were kindly donated by J. Boulter (University of California, Los Angeles) [20], [21] and were transferred to pcDNA3 plasmids (Invitrogen). HEK 293 cells were plated in 35 mm dishes and transfected with 2 µg of each plasmid containing either the α4 or β2 nicotinic acetylcholine receptor subunit and 0.5 µg of a GFP-containing plasmid using lipofectamine (Invitrogen). The day after transfection, cells were trypsinized, re-plated onto 35 mm Petri dishes and used for electrophysiology experiments within 72 hours.

### Electrophysiology

Whole-cell membrane currents were recorded at a membrane potential of –67 mV for neurons and –87 mV for PC12 and HEK 293 cells. Values are corrected for measured liquid junction potentials. The recordings were made with an EPC-7 patch-clamp system (List, Darmstadt, Germany). Currents were low-pass filtered at 3 kHz (8-pole Bessel) and digitized with a LabMaster interface under the control of pClamp software (Axon Instruments). The standard extracellular solution was (in mM) NaCl 165, KCl 5, CaCl_2_ 2, MgCl_2_ 1, D-glucose 10, 2-[4-(2-hydroxyethyl)piperazin-1-yl]ethanesulfonic acid (HEPES) 5, and NaOH ∼2, pH 7.3, with added tetrodotoxin (0.15 µM, Alomone Labs, Israel), when recording in neurons, and atropine sulfate (0.5 µM). This solution replaced the culture medium approximately 20 min before the recordings and was continuously perfused at a rate of ∼1 ml/min throughout the experiments. A U-tube system controlled by the pClamp software was used to deliver fast pulses of drug solutions onto the patch-clamped cells. Unless otherwise noted, fast nicotinic responses were evoked by pulses of acetylcholine chloride dissolved in extracellular solution. Pulse duration was 0.5 s for neurons and PC12 cells, and 2 s for HEK 293 cells. In screening experiments, putative antagonists were applied with acetylcholine, through the U-tube only, but for the inhibition curves they were also pre-applied before acetylcholine via bath solution so that they were in equilibrium at the time of the agonist pulse. Patch micropipettes were made from borosilicate glass capillaries (WPI, Sarasota, FL) in a P-97 horizontal puller (Sutter Instruments, Novato, CA). The intracellular solution was (in mM) CsCl 80, CsF 80, glycol-bis(2-aminoethylether)-N,N,N′,N′-tetraacetic acid (EGTA) 10, HEPES 10, and CsOH ∼6, pH 7.3. The filled patch microelectrodes had resistances of 2 to 5 MΩ in the bath; the access resistance was left uncompensated. Recordings were made at room temperature (23°C).

Peak amplitude and area (charge transfer) were measured for each current trace after baseline subtraction, using pClamp software. These values were then expressed as percent of the controls measured in the same cell. A minimum of three cells were assayed per condition and data are reported as mean ± SEM. Concentration-response data were analyzed by nonlinear regression with the Hill equation, constraining the maxima and minima to 100% and 0%, respectively. The estimated mean inhibitory concentrations (IC_50_) are reported with their 95% confidence interval.

### Alkaloid preparation

Plant material, alkaloid extraction, and isolation were previously described [9]. Stock solutions (0.1 M) were prepared in anhydrous dimethyl sulfoxide (DMSO) and were diluted in standard extracellular solution just before the experiments. Final DMSO concentrations were no greater than 0.1% v/v.

## Results

### Alkaloid screening

**Figure 2:**
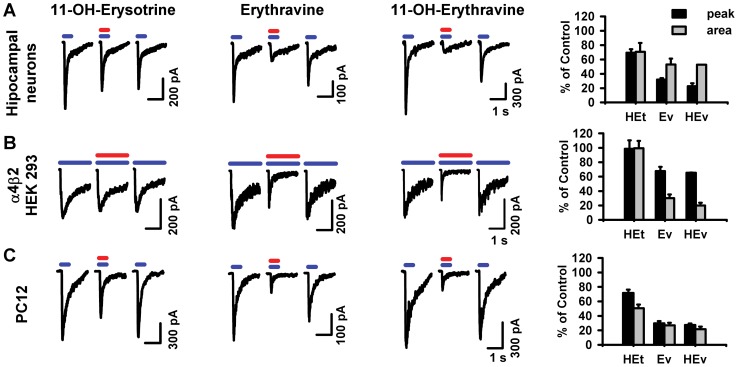
Screening of the *Erythrina* alkaloids. A. Hippocampal neurons constitutively expressing α7* nicotinic receptors. Currents elicited by 0.5-s pulses of acetylcholine 100 µM (blue bars) were partially and reversibly blocked by co-application with 100 µM of (+)-11α-hydroxyerysotrine, (+)-erythravine and (+)-11α-hydroxyerythravine (red bars). Membrane potentials were fixed at –67 mV. **B.** HEK 293 cells heterologously expressing α4β2 nicotinic receptors. Currents elicited by 2-s pulses of acetylcholine 50 µM (blue bars) were partially and reversibly blocked by co-application with 10 µM of (+)-11α-hydroxyerysotrine, (+)-erythravine and (+)-11α-hydroxyerythravine (red bars). Membrane potentials were fixed at –87 mV. **C.** PC12 cells constitutively expressing α3* nicotinic receptors. Currents elicited by 0.5 s pulses of acetylcholine 100 µM (blue bars) were partially and reversibly blocked by co-application with 50 µM of (+)-11α-hydroxyerysotrine, (+)-erythravine and (+)-11α-hydroxyerythravine (red bars). Membrane potentials were fixed at –87 mV. All experiments were performed in the presence of 0.15 µM of TTX (only in experiments with neurons) and 0.5 µM of atropine sulfate. Traces are representative of 3 to 5 independent cells and the mean responses are shown in the bar graphs to the right as the percentages of the current obtained from the first acetylcholine pulse, with error bars being the SEM. Black bars represent the current at peak and grey bars represent the area under the trace for a period of 1.5 s for α4β2 HEK 293 cells and PC12 cells and 1 s for hippocampal neurons, starting at the beginning of the agonist pulse. HEt, (+)-11α-hydroxyerysotrine; Ev, (+)-erythravine; HEv, (+)-11α-hydroxyerythravine.

First the alkaloids were screened for their ability to block nicotinic responses in different preparations, each expressing a major neuronal nicotinic receptor subtype. In all cases we used a near-EC_50_ concentration of acetylcholine and a fixed, high concentration of alkaloids in single application experiments. Hippocampal neurons, which predominantly express α7* nicotinic acetylcholine receptors [19], [22], were stimulated by 100 µM acetylcholine and then concomitantly by acetylcholine and one of the three alkaloids also at 100 µM (Fig. 2A). (+)-Erythravine and (+)-11α-hydroxyerythravine reduced the peak amplitude of acetylcholine-evoked currents to 32.0±2.0% and 22.5±4.4% of control, respectively. The charge carried during the agonist pulse was less reduced than the peak, and the inhibition was mostly reversed after 1 minute. In contrast, (+)-11α-hydroxyerysotrine was less effective and reduced the peak amplitude and charge transfer to similar extents (69.3% and 70.7%, respectively).

Inward currents were evoked by acetylcholine in HEK 293 cells transiently expressing α4β2 nicotinic acetylcholine receptor with an EC_50_ value of 30 µM (18 to 51 µM) and a Hill coefficient of 1.0 (data not shown). As expected, currents elicited by 2-s pulses of acetylcholine 100 µM were completely blocked by 100 nM DHβE, when the cells were pre-exposed to the inhibitor (data not shown). To screen for inhibition, cells were stimulated by 50 µM acetylcholine pulses and then concomitantly by acetylcholine and one of the three alkaloids at 10 µM (Fig. 2B). (+)-Erythravine and (+)-11α-hydroxyerythravine effectively reduced the acetylcholine-evoked currents, with a more pronounced effect on the charge transfer. The peak amplitudes were reduced to 67.7±6.1% and 65.4±0.4% of control, while the areas were down to 30.2±5.1% and 20.1±3.8% of control, respectively. The response recovered completely in the following acetylcholine pulse, without antagonist. (+)-11α-hydroxyerysotrine had no effect on the α4β2 response, at 10 µM.

We next tested whether the alkaloids were able to block nicotinic responses in PC12 cells, a widely used model for the study of neuronal nicotinic acetylcholine receptors. We have not induced further neuronal differentiation of the cells, which were round, adhered lightly to the collagen substrate and presented 2–3 short processes (< 3 µm). In these cells, whole-cell currents elicited by acetylcholine showed an EC_50_ of 46 µM (38 to 57 µM) and were blocked by low concentrations of mecamylamine (1 µM) in a use-dependent manner. Furthermore, neither 100 nM DHβE nor 1 nM methyllycaconitine (an α7* nicotinic acetylcholine receptor antagonist) affected these currents and choline acted as partial agonist (data not shown). These data are typical of responses mediated by ganglionic (α3*) nicotinic acetylcholine receptor with negligible contribution of α4 or α7-containing receptors [23], as previously described for certain populations of PC12 cells [24].

In single application experiments, all three alkaloids (at 50 µM) were able to reduce currents elicited by acetylcholine 100 µM when co-applied with this agonist (Fig. 2C). Again, (+)-erythravine and (+)-11α-hydroxyerythravine had a greater effect than (+)-11α-hydroxyerysotrine. The areas under the traces were reduced to 27.1±3.3%, 21.6±3.6%, and 50.5±5.2% of control, respectively.

### Concentration-response curves

**Figure 3:**
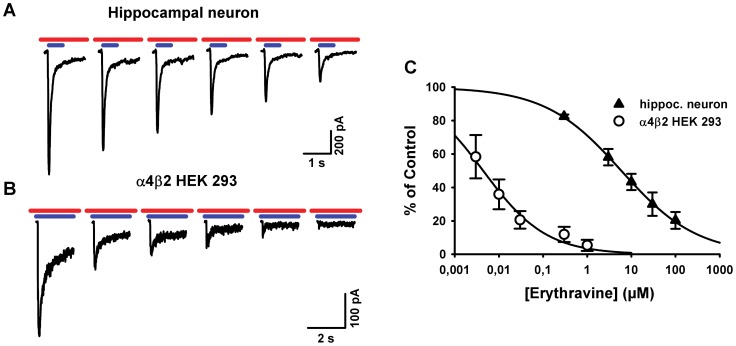
Concentration dependence of the blockade of nicotinic receptor-mediated currents by (+)-erythravine. A. Currents elicited by 2-s pulses of acetylcholine 50 µM (blue bars) in a representative HEK 293 cell expressing α4β2 receptors in the presence of increasing concentrations of (+)-erythravine (0.003 to 1 µM; red bars). **B.** Currents elicited by 0.5 s pulses of acetylcholine 300 µM (blue bars) in a representative hippocampal neuron expressing α7* receptors in the presence of increasing concentrations of (+)-erythravine (0.3 to 100 µM; red bars). The alkaloid was pre-applied on the bathing solution and was in equilibrium during the agonist pulse. **C**. Concentration-response curves showing the area under the current traces obtained as in **A** and **B**. Non-linear regression using the Hill equation yielded an IC_50_ of 4 nM and a Hill coefficient of ­–0.6 for α4β2 receptors (empty circles) and an IC_50_ of 6 µM and a Hill coefficient of ­–0.5 for native α7* receptors (filled triangles). Data are presented as means ± SEM (n  =  3 cells).

The two most potent compounds were then chosen for further quantitative evaluation of their interaction with the α7* and α4β2 nicotinic acetylcholine receptor. For the inhibition curves, each concentration of the alkaloid was applied to the cell by bath perfusion before the agonist pulse, which also contained the alkaloid. Responses were measured as the areas under the current trace. (+)-Erythravine at increasing concentrations progressively inhibited the response to 300 µM acetylcholine in α7-expressing neurons (Fig. 3). Non-linear regression using the Hill equation yielded an IC_50_ value of 5.9 µM (3.9 to 9.0 µM), with a Hill coefficient of 0.50. In HEK 293 cells expressing the α4β2 nicotinic acetylcholine receptor, (+)-erythravine potently inhibited the currents evoked by 50 µM acetylcholine. Inhibition was nearly complete at 1 µM and the IC_50_ value was 4.4 nM (2.1 to 9.4 nM), with a Hill coefficient of 0.61.

**Figure 4:**
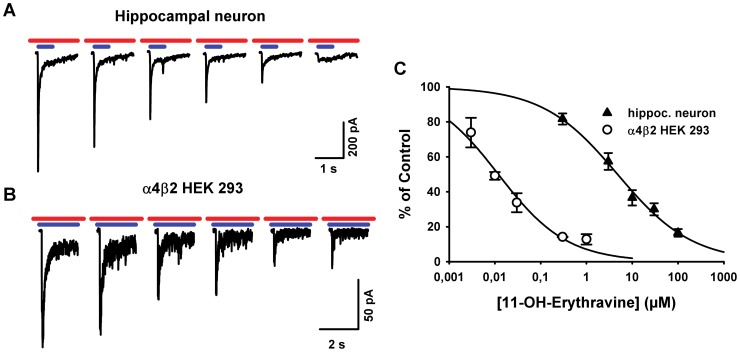
Concentration dependence of the blockade of nicotinic receptor-mediated currents by (+)-11α-hydroxyerythravine. A. Currents elicited by 2-s pulses of acetylcholine 50 µM (blue bars) in a HEK 293t cell expressing α4β2 receptors in the presence of increasing concentrations of (+)-11α-hydroxyerythravine (0.003 to 1 µM; red bars). **B.** Currents elicited by 0.5 s pulses of acetylcholine 300 µM (blue bars) in a hippocampal neuron expressing α7* receptors in the presence of increasing concentrations of (+)-11α-hydroxyerythravine (0.3 to 100 µM; red bars). The alkaloid was pre-applied on the bathing solution and was in equilibrium during the agonist pulse. **C**. Concentration-response curves showing the area under the current traces obtained as in **A** and **B**. Non-linear regression using the Hill equation yielded an IC_50_ of 12 nM and a Hill coefficient of ­–0.6 for α4β2 receptors (empty circles) and an IC_50_ of 5 µM and a Hill coefficient of ­–0.5 for native α7* receptors (filled triangles). Data are presented as means ± SEM (n  =  3 cells).

The hydroxylated analogue (+)-11α-hydroxyerythravine inhibited the response to acetylcholine in α7-expressing neurons (Fig. 4) with a similar IC_50_ value of 4.8 µM (3.4 to 6.7 µM), and a Hill coefficient of 0.53. In HEK 293 cells expressing the α4β2 nicotinic acetylcholine receptor, (+)-11α-hydroxyerythravine also potently inhibited acetylcholine-stimulated currents, with an IC_50_ value of 11.9 nM (7.7 to 18.3 nM) and Hill coefficient of 0.58.

## Discussion

Our data demonstrate that the alkaloids from *E. mulungu* previously shown to have anxiolytic-like effects in rodents inhibit neuronal nicotinic acetylcholine receptors. Although the two main receptor subtypes expressed in the mammalian brain were sensitive to the alkaloids, the α4β2 receptor was more potently inhibited. Because low nanomolar concentrations of (+)-erythravine and (+)-11α-hydroxyerythravine markedly inhibited the α4β2 receptor function, this receptor must be considered a possible target of phytotherapeutic preparations of *E. mulungu*.

In spite of the high potency of (+)-erythravine and (+)-11α-hydroxyerythravine, other compounds may contribute to the α4β2 receptor-dependent effects of *E. mulungu* extracts. Santos Rosa and collaborators [10] recently isolated erysotrine from the flowers of *E. mulungu* and demonstrated a marked anticonvulsant and a limited anxiolytic-like effect of this alkaloid (i.c.v.) in rats. Based on negative results in binding and uptake assays in synaptosomes, the authors excluded mechanisms involving GABA or glutamate signaling. In fact, erysotrine inhibits currents mediated by α4β2 and α7 nicotinic receptors expressed in *Xenopus* oocytes with IC_50_ of 0.37 µM and 17 µM, respectively [16]. Thus, erysotrine could also target α4β2 receptors to induce its CNS effects, further supporting a role of these receptors in the effects of alkaloid-containing extracts of *E. mulungu*.

Several erythrinic alkaloids are known to be competitive antagonists of CNS nicotinic receptors [16]. An alternative mechanism of fast inhibition of nicotinic receptors is open channel block, which tends to accelerate the decay kinetics of receptor-gated currents. Although we have not investigated the inhibitory mechanisms, we note that (+)-erythravine and (+)-11α-hydroxyerythravine could obliterate the nicotinic receptor currents without obviously affecting their decay rates (data not shown). Therefore, competitive antagonism seems to be the best hypothesis of inhibitory mechanism.

Extracts of *Erythrina* species have been known to induce neuromuscular blockade for a long time, at least since the report by Dominguez and Altamirano in 1877 (cited in [25]) and many erythrinian alkaloids have been isolated and investigated as possible curare substitutes [25]–[28]. Erysodine was one of the first of these curarizing alkaloids to be later characterized as having selectivity toward CNS nicotinic acetylcholine receptor subtypes [29]. The best known erythrinian alkaloid, DHβE, is an effective antagonist of muscle nicotinic acetylcholine receptors, but is now widely used as a pharmacological tool both in vitro and in vivo due to its higher affinity for the α4β2 nicotinic receptor subtype [17]. Pre-treatment with DHβE reduces nicotine self-administration and inhibits nicotine-induced reinforcement of operant behavior in rats, at doses that do not impair motor activity [30], [31]. Similarly, doses of crude *Erythrina mulungu* extract or its isolated alkaloids that induce behavioral effects in rodents are not associated with overt motor impairment [3], [4], [8], [9], [32], [33]. Therefore, if (+)-erythravine and (+)-11α-hydroxyerythravine also inhibit muscle-type (α1_2_β1εδ) nicotinic receptors, they are likely to be much less potent than what we have found for α4β2 receptors.

The actual mechanism by which nicotinic acetylcholine receptors affect anxiety is controversial but available evidence suggests the involvement of 5-hydroxytryptamine receptors [14], [34]. Different brain regions and nicotinic acetylcholine receptor subtypes participate in this phenomenon. Stereotaxic application of nicotine in the raphé dorsal nucleus has anxiolytic-like effects in rats which are blocked by low doses of DHβE [11]. Loss of function of α4β2 nicotinic receptors exclusively in dopaminergic neurons leads to a decreased sensitivity to the anxiolytic effects of nicotine in mice [35]. On the other hand, nicotine application in the dorsal hippocampus has anxiogenic effects that are blocked by methyllycaconitine, suggesting the participation of α7* nicotinic acetylcholine receptors [36]. Nicotine applications to central amygdala also induce an increase in anxiety-like behavior [37]. In addition to these contrasting local effects, the behavioral changes induced by nicotinic modulators given systemically are also complex due to time-dependent effects [38], [39]. Both α4β2 and α7* nicotinic acetylcholine receptors may promote anxiety in the rat, since pre-application of DHβE inhibits nicotine-induced anxiety behaviors and methyllycaconitine itself has anxiolytic effects [15]. Knockout mice for Lynx2, a mammalian prototoxin that diminishes nicotinic acetylcholine receptor function, present enhanced anxiety-related behavior [40] and knockouts for the β3 [41] and β4 [42] nicotinic acetylcholine receptor subunits lead to a decrease in anxiety behavior. Collectively, these data support a role for nicotinic acetylcholine receptor in anxiety.

The mounting evidence of the involvement of nicotinic receptors in anxiety and depression pathways has prompted the search for novel tools to investigate them, as well as for new compounds with potential clinical use. The non-selective stereoisomer *S*-(+)-mecamylamine (TC-5214) has reached phase III clinical trial for concomitant use with citalopram in patients unresponsive to citalopram alone [43], [44], However, its development was discontinued after no significant differences were found when compared to placebo groups. The demonstration that at least two *E. mulungu* alkaloids are potent antagonists of α4β2 nicotinic receptors provides a strong working hypothesis for the mechanism of action of the plant extracts, traditionally used to treat insomnia and agitation. Our data also imply that bioassays for neuronal nicotinic receptor antagonist activity could be used to assess the pharmacological activity of the extracts, providing a rational basis for product standardization and for dosing recommendations.
